# Criminal recidivism in offenders with and without intellectual disability sentenced to forensic psychiatric care in Sweden—A 17-year follow-up study

**DOI:** 10.3389/fpsyt.2022.1011984

**Published:** 2022-09-21

**Authors:** Hanna Edberg, Qi Chen, Peter Andiné, Henrik Larsson, Tatja Hirvikoski

**Affiliations:** ^1^Paediatric Neuropsychiatry Unit, Department of Women’s and Children’s Health, Centre for Neurodevelopmental Disorders at Karolinska Institutet (KIND), Karolinska Institutet, Stockholm, Sweden; ^2^Swedish Prison and Probation Services, Norrköping, Sweden; ^3^Northern Stockholm Psychiatric Clinic, Region Stockholm, Stockholm, Sweden; ^4^Centre for Psychiatry Research, Region Stockholm, Stockholm, Sweden; ^5^Department of Medical Epidemiology and Biostatistics, Karolinska Institutet, Stockholm, Sweden; ^6^Centre for Ethics, Law and Mental Health (CELAM), Institute of Neuroscience and Physiology, Sahlgrenska Academy, University of Gothenburg, Gothenburg, Sweden; ^7^Forensic Psychiatric Clinic, Sahlgrenska University Hospital, Gothenburg, Sweden; ^8^Department of Forensic Psychiatry, National Board of Forensic Medicine, Gothenburg, Sweden; ^9^School of Medical Sciences, Örebro University, Örebro, Sweden; ^10^Habilitation & Health, Region Stockholm, Stockholm, Sweden

**Keywords:** intellectual disability, criminal recidivism, forensic psychiatry, neurodevelopmental disorder, crime, offender, survival analysis

## Abstract

**Background:**

Offenders with intellectual disability (ID) constitute a distinct subgroup of offenders with mental disorders. Regarding criminal recidivism, it is unclear whether or not offenders with ID in forensic psychiatric settings differ from offenders without ID. Factors associated with criminal recidivism among offenders with ID have been scarcely investigated.

**Aim:**

To investigate the association between ID and criminal recidivism among offenders sentenced to forensic psychiatric care and to explore the impact of clinical, sociodemographic and offense variables.

**Materials and methods:**

We conducted a retrospective cohort study based on Swedish nationwide registers. A total of 3,365 individuals being sentenced to forensic psychiatric care in Sweden in 1997–2013 were followed from the forensic psychiatric assessment until first reconviction, death, emigration, or 31 December 2013, whichever occurred first. Cox regression models compared rates of recidivism in individuals with and without ID. Impact of clinical, sociodemographic, and offense variables on risk of criminal recidivism was presented as hazard ratios (HRs) with 95% confidence intervals (CIs).

**Results:**

Out of 3,365 offenders sentenced to forensic psychiatric care, 259 (7.7%) were diagnosed with ID. During follow-up (0–17 years, median 6 years), one third (*n* = 1,099) of the study population relapsed into criminality, giving a recidivism rate of 50.5 per 1,000 person-years. We observed an association between ID and a decreased risk of recidivism (HR 0.8, 95% CI 0.6–1.0, *p* = 0.063), although this reached statistical significance only for the subgroup of male offenders (HR 0.8, 95% CI 0.6–1.0, *p* = 0.040) and not females (HR 1.0, 95% CI 0.6–1.8). ID offenders with concurrent ADHD tended to have a higher rate of recidivism (73.9 per 1,000 person-years, HR 1.2, 95% CI 0.6–2.4) than ID offenders without ADHD (42.5 per 1,000 person-years, HR 0.8, 95% CI 0.6–1.1). Amongst ID offenders, concurrent autism spectrum disorder, young age or male sex were not associated with recidivism, while previous criminal convictions were strongly associated with recidivism.

**Conclusion:**

A diagnosis of ID was associated with a lower risk of criminal recidivism among male offenders sentenced to forensic psychiatric care. The association between ADHD and recidivism among ID offenders highlights eligible focus areas in the management of offenders with ID.

## Introduction

Intellectual disability (ID) is a neurodevelopmental disorder characterized by early onset, significant impairment of intellectual functioning and adaptive behavior (1, 2), affecting about 1% of the general population (3, 4). Comorbid neurodevelopmental disorders such as ADHD (attention-deficit/hyperactivity disorder) and ASD (autism spectrum disorder) are common among individuals with ID (5, 6). Individuals with ID have been reported as overrepresented in the criminal justice system, but base rates of criminal offending among individuals with ID have been difficult to establish, partially because of inconsistent definitions of ID. Prevalence estimates have ranged from well below to above and beyond that of the general population (7). In Sweden, offenders with severe mental disorders can be sentenced to forensic psychiatric care instead of prison (8). The court can order a forensic psychiatric assessment (FPA) to obtain a multi-professional judgment if an alleged offender suffers from a severe mental disorder. A severe mental disorder is a judicial concept that includes mental, behavioral, or emotional conditions that cause serious functional impairment, such as, under certain circumstances, ID (9). In a forensic psychiatric context, offenders with ID differ from their non-ID counterparts in aspects of clinical and sociodemographic characteristics and criminal behavior; they are younger, less socially established, show a higher frequency of concurrent neurodevelopmental disorders and are more likely to have committed a sexual offense (10–13). Whether offenders with ID also differ from non-ID offenders regarding recidivism rate has not been thoroughly investigated. Similarly, risk factors for re-offending are not well-known in this group.

In their most recent meta-analysis, Bonta et al. noted that intellectual impairment was weakly associated with increased risk of recidivism (14). However, the studies that generated this conclusion were published in the 1980–1990s, a period in which the definition of ID differed from contemporary diagnostic criteria (15, 16). In addition, several of the included studies focused exclusively on arsonists. Furthermore, a study by Gray et al. from 2007, which reported slower recidivism rates among offenders with ID (according to ICD-10, codes F70-79), compared to other offenders with mental disorders (17), was included in the meta-analysis, but later omitted from the analysis as an outlier. Several more recent studies on criminal recidivism rates in populations of offenders with mental disorders do not include ID (18–20). In studies where data on ID or intelligence has been included, study samples have been small (21) or only presented data on selected samples, such as subjects sentenced to community services (22, 23). Hence, it remains uncertain whether or not ID is associated with criminal recidivism among offenders with mental disorders.

Several studies have tried to define risk factors for offending among individuals with ID (24–26), often by describing characteristics of offenders with ID and making comparisons to a non-ID or a non-offender group. A latent class analysis of offenders with mental disorders showed that offenders with ID exhibit particular levels of risk and protective factors for criminal behavior that differentiate them from offenders with other mental disorders, meriting distinction as one of five separate groups. Offenders with ID had the lowest levels of protective factors such as insight, coping, and social skills, as well as treatment compliance (27). Furthermore, comorbid ADHD has been suggested to be associated with increased risk of violent offending among individuals with ID (28). However, when studying risk factors for criminal behavior among individuals with ID, it is important to distinguish between risk factors for offending and risk factors for re-offending. While variables such as externalizing behavioral problems and comorbid mental disorders might be associated with increased risk of offending among individuals with ID (25, 29–31), the same factors might hypothetically prompt an augmented level of support and more extensive rehabilitation measures following a criminal act, which can in turn reduce the risk of re-offending. In Sweden for example, individuals with ID are subject to specific legislation to ensure adequate support (32). In order to receive these services, the individual must be known by the social authorities. A contact with the criminal justice system might be the factor that draws attention to the individual, which initiates support. Measures such as personal assistance, financial support, counseling, adapted housing and daily activities are granted according to the individual need. The greater the need the more extensive the measures of support. Factors associated with first time offending, might therefore induce social support acting as preventive measures on risk of re-offending.

Previous meta-analyses (14, 33) and more recent studies (21, 34) suggest that the strongest predictors of criminal recidivism among offenders with mental disorders are criminogenic variables (i.e., criminal history and deviant lifestyle including substance abuse and antisocial behavior); far exceeding the predictive value of potential clinical variables such as psychiatric comorbidity, prior hospital admissions and psychiatric treatment. A more limited number of studies have focused specifically on factors related to criminal recidivism specifically in ID. A study by Lofthouse et al. bears references to re-offending, by describing how different risk factors can predict future violent behavior (not specifically reconvictions) among offenders with ID. The authors suggest that even though static risk factors (unchangeable factors such as age at first conviction and previous criminal convictions) dominate dynamic factors (e.g., behavioral aspects or clinical symptoms that are amenable to change) in the prediction of the risk of future violent behavior among offenders with ID, dynamic variables serve as proxy risk factors for static risk and are therefore both useful and important in assessment (35). Fitzgerald analyzed the predictive effect of criminal history variables and substance use variables on criminal reconviction among individuals with ID in medium secure hospitals (individuals who had offended, or who had exhibited behavior that might have led to a conviction under different circumstances) and found that criminal history and substance use variables predicted future reconviction in offenders with ID, much the same as among other offenders (36). Marti-Augusti et al. conducted a review on offenders with ID (37), including data on both offending and recidivism. They suggested that in addition to known risk factors, well-described by Bonta et al. (14, 33), clinical variables such as personality disorders were risk factors for reoffending among offenders with ID. However, a number of the referred studies included borderline intellectual functioning, currently not included in the definition of ID (38–40), or were reviews based on studies going back 20–30 years in time (41, 42). Study samples dating back several decades can be problematic since there has been a marked increase of diagnoses of ADHD and ASD during the last 30 years (43, 44), supposedly related to changes in diagnostic practices (45, 46).

To conclude, whether or not ID is associated with criminal recidivism among offenders with mental disorders is unclear. Therefore, the primary aim of the study is to investigate if ID is associated with increased or decreased risk of criminal recidivism among offenders with mental disorders who have been sentenced to forensic psychiatric care. There is a lack of research regarding risk factors for criminal recidivism among offenders with ID. Concurrent neurodevelopmental disorders such as ADHD and ASD among offenders with ID are a potentially tangible intervention target and hence a research area that merits more attention (39, 47). Hence, the secondary aim of the study is to study the effect of clinical, sociodemographic, and offense variables on criminal recidivism among offenders with ID, compared to offenders with other mental disorders.

## Materials and methods

### Study design

We conducted a population-based cohort study of individuals sentenced to forensic psychiatric care in Sweden using data from the Swedish national registers.

### Data

Our data originated from the Central Archive of the National Board of Forensic Medicine, where data from all FPAs in Sweden are registered. Data was pseudonymized and linked to national population-based registers.

### Study population

The cohort included 3,365 individuals who were sentenced to forensic psychiatric care after FPA in Sweden between 1 January 1997 and 30 May 2013. During the study period, a total of 8,442 individuals were subject to FPA. All individuals for whom it was possible to identify an index offense were included, which comprised 88% (*n* = 7,450) of the total population. As described in previous work (13), there were no significant differences in age, sex, psychiatric diagnoses, parental education level, or immigration status between the total source population of 8,442 individuals and the individuals included in the study.

### Study setting

Individuals were followed from the date when the result of the FPA was presented to the court until first reconviction date (criminal recidivism), death, emigration from Sweden or 31 December 2013, whatever occurred first. The start of follow-up was chosen since it has been shown that up to 10 percent of individuals sentenced or diverged to forensic psychiatric care commit a new criminal offense whilst under forensic psychiatric care (21, 48–50). The duration of follow-up was up to 17 years (median 6 years).

### Exposures

Exposure in the primary analysis was a diagnosis of ID, registered during the FPA. The diagnosis derives from a thorough team-based medical-psychiatric assessment with a psychological assessment of intellectual and adaptive functioning including evaluation using the Swedish version of the Wechsler Adult Intelligence Scale (51). Diagnoses were coded according to the Diagnostic and Statistical Manual of Mental Disorders, 4th version (DSM-IV) (51). DSM-IV codes for ID included 317, 318.0, 318.1, 318.2, 319. Exposure in the secondary analysis was clinical variables (i.e., concurrent psychiatric diagnoses). Sociodemographic variables and offense variables were treated as exposures in the third analysis.

### Outcomes

The outcome was defined as first criminal reconviction registered by the court. Main analyses included reconviction of any offense as outcome. Descriptive statistics included sub-categories of crime, including violent non-sexual, sexual and non-sexual non-violent crime.

### Covariates

#### Sociodemographic variables

Sociodemographic data were assessed at the time of the FPA, i.e., right before start of follow-up. Data derived from Swedish population-based registers, including the longitudinal integration database for health insurance and labor market studies covering all Swedish residents ≥ 16 years of age (52), the Multi-Generation Register and the Total Population Register (53). Variables included age (at the time of the FPA if not otherwise specified), sex, immigration status (born in or outside Sweden) and highest parental educational level (<9 years, 9 years, >9 years).

#### Clinical variables

Clinical variables included psychiatric diagnoses derived from the FPA, coded according to the DSM-IV. Concurrent psychiatric diagnoses included ADHD, ASD, alcohol use disorders, drug use disorders, personality disorders (including antisocial personality disorders, borderline personality disorders, and other personality disorders), schizophrenia, and sexual disorders. Diagnostic codes can be found in Supplementary Table 1.

#### Offense variables

Offense variables included index crime type (crime resulting in sentence to forensic psychiatric care) and previous crime (yes/no). Criminal offenses were categorized according to the Swedish Penal Code in three categories: violent non-sexual, sexual and non-sexual non-violent. Index crimes were obtained from the Central Archive of the National Board of Forensic Medicine. The Swedish National Council for Crime Prevention (Brå) provided data regarding previous convictions and reconvictions during follow-up. A thorough description of the rationale behind the offense variables can be found in previous work (13).

### Statistical analyses

Descriptive characteristics for ID and non-ID offenders at baseline were reported as percentages for categorical variables and medians with interquartile range (IQR) for continuous variables. We constructed cumulative incidence curves for first reconviction for any offense among offenders with and without ID using the Kaplan-Meier method with Log-Rank test. Separate analyses were not performed on sub-categories of crimes because of the limited sample size. The association between ID and rate of recidivism was analyzed using the Cox regression model estimating hazard ratio (HR) with 95% confidence interval (CI). In a sensitivity analysis, the model was stratified by sex.

In order to study the effect of clinical variables (i.e., concurrent psychiatric diagnoses) on the risk of recidivism among ID offenders and non-ID offenders, we calculated the incidence rates of recidivism in different subgroups of psychiatric disorders and used Cox regression analyses to estimate HRs, using individuals without ID and without the clinical variable as the reference group.

In the analysis of association between sociodemographic and offense variables, and recidivism among ID offenders and non-ID offenders, HRs were estimated. Parental educational level was missing in 34.7% of cases. Under the assumption of missing at random, we handled the missing data by applying multiple imputation, with 20 imputed samples (54). We analyzed each imputed dataset and reported the pooled estimates.

All Cox proportional hazard models were adjusted for age, sex, previous convictions, and parental educational level. In the analysis of sociodemographic and offense variables, adjustment was also made for concurrent psychiatric diagnoses. The proportionality of hazard assumption was tested by measuring interactions between covariates and time. Statistical analyses were conducted using the IBM SPSS Statistics 28 software. All statistical tests were two-tailed with *p*-value < 0.05 considered significant.

## Results

Characteristics of the population are presented in Table 1, for ID (*n* = 259) and non-ID (*n* = 3,106) offenders. Follow-up time was 0–17.5 years (median 6 years). Out of 3,365 individuals, a total of 1,099 (33%) were convicted of at least one new crime during follow up. Individuals with ID presented slightly lower crude rates of recidivism at end of follow-up than individuals without ID (29 vs. 33%).

**TABLE 1:** Characteristics of offenders with and without ID sentenced to forensic psychiatric care in Sweden in 1997–2013 (*n* = 3,365).

	ID (*n* = 259)	Non-ID (*n* = 3106)
**Sociodemographic variables n (%)**		
Male	205 (79.2)	2,637 (84.9)
Female	54 (20.8)	469 (15.1)
Median age, years (IQR)	29 (17)	36 (18)
Born in Sweden	190 (73.4)	2,085 (67.1)
***Parental educational level***		
<9 years^a^	56 (29.6)	456 (22.7)
≥9 years	133 (70.4)	1,553 (77.3)
**Offense variables n (%)**		
Previous conviction	185 (71.4)	2,404 (77.4)
Reconviction	74 (28.6)	1,025 (33.0)
***Index crime category***		
Violent non-sexual	188 (72.6)	2,628 (84.6)
Sexual	58 (22.4)	248 (8.0)
**Clinical variables n (%)**		
ADHD	21 (8.1)	123 (4.0)
ASD	66 (25.5)	313 (10.1)
Alcohol use disorder	50 (19.3)	627 (20.2)
Drug use disorder	27 (10.4)	949 (30.6)
Personality disorder^b^	55 (21.2)	817 (26.3)
Schizophrenia	17 (6.6)	929 (29.9)
Sexual disorder	15 (5.8)	76 (2.4)

IQR, Interquartile range; ADHD, Attention Deficit Hyperactivity Disorder; ASD, Autism Spectrum Disorder.

^a^Highest parental educational level and total years of education.

^b^Any personality disorder.

### Association between ID and criminal recidivism

**FIGURE 1:**
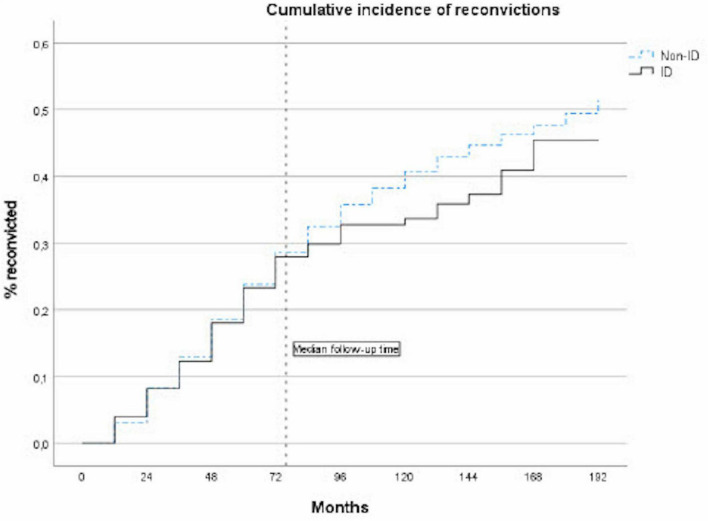
Cumulative incidence of reconvictions, comparing ID and non-ID offenders sentenced to forensic psychiatric care.

Cumulative incidence curves for first reconvictions during a follow-up are presented in Figure 1. Offenders with ID tend to relapse into crime at a slower pace than offenders without ID after 7 or more years following a sentence to forensic psychiatric care, however, the overall difference did not reach statistical significance (log rank test 1.209, *p* = 0.272).

In the entire study population, after adjustment for age, sex, previous criminal convictions, and parental educational level, we observed an association between ID and decreased risk of criminal recidivism (HR 0.8, 95% CI 0.6–1.0), although this did not reach statistical significance (*p* = 0.063) (Table 2). Sex stratified analyses showed that the association was significant among male offenders (HR 0.8, 95% CI 0.6–1.0, *p* = 0.040), but not among female offenders (HR 1.0, 95% CI 0.6–1.8) (Supplementary Table 2).

**TABLE 2:** Estimated hazard ratios for reconvictions, comparing ID and non-ID offenders, adjusted for potential confounders (age, sex, previous convictions, and parental educational level).

Offenders sentenced to forensic psychiatric care (*n* = 3,365)	Any criminal reconviction			

**Exposure category**	**Events**	**Person years**	**Incidence rate^a^ (95% CI)**	**Hazard ratio (95% CI)**
ID (*n* = 259)	74	1,660	44.6 (44.3–44.9)	0.8 (0.6–1.0)
Non-ID (*n* = 3,106)	1,025	20,101	51.0 (50.9–51.1)	Reference

^*a*^Per 1,000 person years.

Reconviction categories are presented in Table 3. Violent non-sexual reconvictions were more common among ID than among non-ID offenders (35 vs. 24%), non-sexual non-violent reconvictions were more common among non-ID offenders (75 vs. 62%) and sexual reconvictions were uncommon in both groups (2.7% among ID offenders, 1.8% among non-ID offenders).

**TABLE 3:** Reconvictions at end of follow-up among offenders with and without ID.

	Total (*n* = 3,365)	ID (*n* = 259)	Non-ID (*n* = 3,106)
**Reconvictions (% of n)**			
All crimes n (%)	1,099 (32.7)	74 (28.6)	1,025 (33.0)
**Subcategories (% of all reconvictions)**			
Non-sexual non-violent n (%)	811 (73.8)	46 (62.2)	765 (74.6)
Violent non-sexual n (%)	268 (24.4)	26 (35.1)	242 (23.6)
Sexual n (%)	20 (1.8)	2 (2.7)	18 (1.8)

### Association between clinical variables and criminal recidivism

**FIGURE 2:**
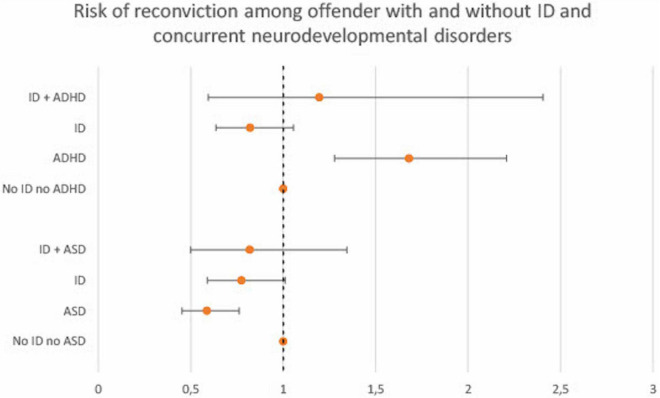
Cox proportional hazard ratios (with 95% CI) for criminal recidivism associated with clinical variables among offenders with and without ID. Reference category for each group are offenders with neither ID nor the clinical variable (ADHD and ASD, respectively). ADHD, Attention Deficit Hyperactivity Disorder; ASD, Autism Spectrum Disorder.

**FIGURE 3:**
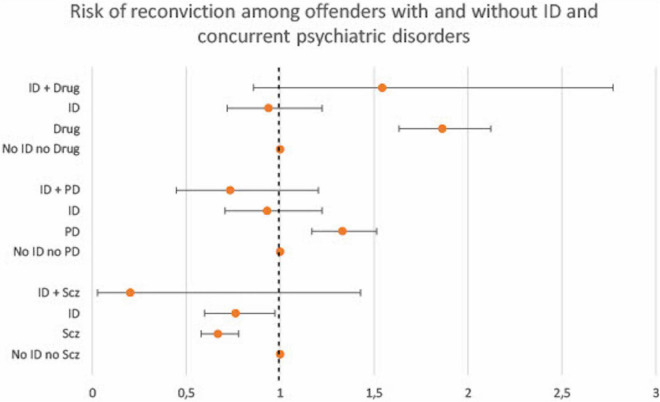
Cox proportional hazard ratios (with 95% CI) for criminal recidivism associated with clinical variables among offenders with and without ID. Reference category for each group are offenders with neither ID nor the clinical variable (drug use disorder, personality disorder or schizophrenia, respectively). Drug, Drug use disorder; PD, Any personality disorder; Scz, Schizophrenia.

Results regarding clinical variables are presented in Figures 2, 3 (full data can be found in Supplementary Table 3). Sexual disorders were omitted because of small actual numbers (total cases < 25). Alcohol use disorders showed no association to recidivism and did not differ between ID and non-ID offenders, and was thus omitted from the figure. In both groups, schizophrenia was associated with decreased recidivism risk (ID and schizophrenia: HR 0.2, 95% CI 0.0–1.4; non-ID and schizophrenia: HR 0.7, 95% CI 0.6–0.8). In both groups, increased risk was observed in association with drug use disorders (ID and drug use disorders: HR 1.5, 95% CI 0.9–2.8; non-ID and drug use disorders: HR 1.9, 95% CI 1.6–2.1) and ADHD (ID and ADHD: HR 1.2, 95% CI 0.6–2.4; non-ID and ADHD: HR 1.7, 95% CI 1.3–2.2). In subjects with ID, data could not support an effect of concurrent ASD or personality disorders on recidivism risk (ID and ASD: HR 0.8, 95% CI 0.5–1.3; ID and personality disorders: HR 0.7, 95% CI 0.4–1.2). In subjects without ID, ASD was associated with decreased recidivism risk (non-ID and ASD: HR 0.6, 95% CI 0.5–0.8) and personality disorders with increased recidivism risk (non-ID and personality disorders: HR 1.3, 95% CI 1.2–1.5).

### Association between sociodemographic and offense variables and criminal recidivism

The final analysis evaluated the association between sociodemographic and offense variables and criminal recidivism, comparing offenders with and without ID. Results are presented in Table 4. Previous criminal convictions showed the strongest association with increased risk of criminal recidivism in both offenders with and without ID (ID and previous convictions: HR 3.0, 95% CI 1.4–6.1; non-ID and previous convictions: HR 1.9, 95% CI 1.5–2.3). Among subjects with ID, being male was not associated with risk of recidivism (ID and male: HR 1.1, 95% CI 0.6–2.0), which was the case among subjects without ID (non-ID and male: HR 1.4, 95% CI 1.1–1.7). Among subject without ID, the youngest age group (age 15–24) was associated with the highest risk of recidivism (non-ID and age 15–24: HR 3.3, 95% CI 2.3–4.8) and the risk decreased gradually with increased age. Among subjects with ID, no obvious association between age and criminal recidivism was seen. The risk did not decrease gradually with age and was equally high among 15–24-year-olds (ID and age 15–24: HR 1.5, 95% CI 0.2–13.0) as among 35–44-year-olds (ID and age 35–44: HR 1.5, 95% CI 0.2–11.9). Being born in Sweden was associated with lower risk of recidivism among subjects with ID (ID and born in Sweden: HR 0.6, 95% CI 0.4–1.0), but did not affect the risk among subjects without ID (non-ID and born in Sweden: HR 1.0, 95% CI 0.6–1.1). Low parental educational level was associated with an increased risk of recidivism among subjects with ID (ID and low parental level: HR 1.6, 95% CI 0.7–3.5) but not among subjects without ID (non-ID and low parental level: HR 1.2, 95% CI 1.0–1.4).

**TABLE 4:** Cox proportional hazard ratios for reconvictions associated with sociodemographic and offense variables comparing offenders with and without ID.

	Offenders with ID (*n* = 259)		Offenders without ID (*n* = 3,106)	
	HR, univariate analysis (95% CI)	HR, multivariate analysis^a^ (95% CI)	HR, univariate analysis (95% CI)	HR, multivariate analysis^a^ (95% CI)
**Sociodemographic variables n (%)**				
Male	1.0 (1.0–1.8)	1.1 (0.6–2.0)	1.5 (1.3–1.9)	1.4 (1.1–1.7)
Female	Reference		Reference	
*Age group*				
15–24	2.6 (0.3–19.2)	1.5 (0.2–13.0)	3.3 (2.3–4.6)	3.3 (2.3–4.8)
25–34	2.3 (0.3–17.0)	1.0 (0.1–8.4)	3.1 (2.2–4.4)	3.0 (2.1–4.2)
35–44	3.1 (0.4–23.2)	1.5 (0.2–11.9)	2.9 (2.0–4.0)	2.7 (1.9–3.8)
45–54	2.2 (0.3–17.4)	1.0 (0.1–8.7)	1.8 (1.3–2.6)	1.7 (1.2–2.5)
55+	Reference		Reference	
Born in Sweden	0.6 (0.4–1.0)	0.6 (0.4–1.0)	1.0 (0.8–1.1)	1.0 (0.6–1.1)
Born outside Sweden	Reference		Reference	
*Parental educational level*				
<9 year	1.3 (0.7–2.5)	1.6 (0.7–3.5)	0.9 (0.8–1.1)	1.2 (1.0–1.4)
9 years	1.2 (0.5–3.0)	1.4 (0.6–3.5)	1.0 (0.8–1.4)	1.0 (0.7–1.4)
>9 years	Reference		Reference	
**Offense variables n (%)**				
Previous conviction	3.4 (1.7–6.8)	3.0 (1.4–6.1)	2.3 (1.9–2.8)	1.9 (1.5–2.3)
No previous conviction	Reference		Reference	

Univariate and multivariate analyses. HR, hazard ratio; ADHD, Attention Deficit Hyperactivity Disorder; ASD, Autism Spectrum Disorder.

^a^Including age, sex, previous criminal convictions, parental educational level, and concurrent clinical diagnoses (schizophrenia, personality disorders, ADHD, ASD, drug use, alcohol use, and sexual disorder).

## Discussion

In this Swedish nationwide register-based cohort study of 3,365 offenders sentenced to forensic psychiatric care, we observed an association between ID and decreased risk of criminal recidivism after adjustment for eligible confounders. This finding is in concordance with the results from Gray et al. who reported that ID individuals reoffended at a significantly lower rate than their non-ID counterparts (17). By contrast, our results can be seen as contradictory to the meta-analysis by Bonta et al. (14), who reported an increased risk of criminal recidivism among offenders with intellectual impairment. Bonta et al. did however employ the concept of “intellectual impairment” in their study, which can include, but is not necessarily synonymous with, a diagnosis of ID. This highlights the importance of distinguishing ID from borderline ID and using clear and transparent definitions in research on ID offenders.

Our results could only verify a risk reduction related to ID among male offenders (constituting over 80% of the current study population). No obvious association between ID and recidivism was observed among females. Future research on a larger sample of female offenders is needed to further investigate the relationship between ID and recidivism.

The impact of psychiatric diagnoses on criminal recidivism risk showed both similarities and discrepancies among offenders with and without ID. ADHD was associated with increased recidivism risk in subjects without ID and a similar tendency was seen in subjects with ID, although not reaching statistical significance. This finding is in line with results from a Swedish cohort study, where the elevated risk of violent offending among ID individuals to a great extent was explained by comorbid ADHD (28). Our data thus suggest that the same is true regarding the risk of reoffending. These findings bear great importance, since ADHD can be successfully treated both in general and offender populations (55, 56) and treatment has been associated with decreased risk of criminal recidivism among offenders (57). Assessment and treatment of concurrent ADHD among ID offenders should be an integrated part of individualized care plans in forensic psychiatric settings.

Concurrent ASD did not affect the recidivism risk in any direction in subjects with ID. Among subjects without ID, however, ASD was associated with decreased risk of criminal recidivism. These are important results since there is limited research on the impact of ASD on offending behaviors among ID offenders (47).

Among offenders with ID, young age and being male were not variables associated with greater risk of reconviction. This finding can be interpreted as a feature distinguishing ID offenders from other offenders with mental disorders (21, 33, 34) and from general adult offenders (58), where being young and male is typically associated with a greater risk of recidivism. However, a recent meta-analysis of 28 studies investigating predictors of criminal recidivism among forensic outpatients did not replicate the findings of Bonta el al. and Gendreau et al. regarding age and sex as risk factors for recidivism (59). Sex was also not associated with recidivism in a study on 315 patients discharged from a medium-secure hospital in the UK (60). In a recent study of 477 offenders with mental disorders by Dean et al. results suggested that while sex was not associated with reconviction rates overall, women reoffended to a higher degree than men during the first 12 months following release (61). Sex differences in criminal recidivism studies in populations of offenders with mental disorders thus present diverging results. Besides offenders with mental disorders being heterogeneous populations making comparisons difficult, another plausible interpretation is that while male sex used to show strong association with criminal recidivism, sex differences in criminal behavior have started to level during the last decades (62): a phenomenon described as a “narrowing gender gap” (63, 64). Our findings add important knowledge to the field, since a number of previous studies on recidivism in ID offenders have not been able to study sex differences due to restricted sample size (36) or sample selection (65). Opposite to our results, young age was suggested as a risk factor for recidivism in a previous study on 67 ID offenders (66). Considering that the vast majority of studies of recidivism in ID offenders suffer from lack of statistical power, each contribution is of importance to increase the knowledge in the field.

More research is needed in order to develop risk assessment instruments with adequate predictive ability. While a number of studies have suggested that well-studied static risk assessment instruments such as the Violence Risk Appraisal Guide (VRAG) (67), HCR-20 (68) and the Psychopathy Checklist (PCL-R) (69, 70) can be used to predict violence and reconvictions among ID offenders (17, 24, 36), these instruments were not developed for use in ID populations, and might therefore lack variables that can be predictive specifically in these individuals. Our study presents data on associations between ID, clinical, sociodemographic and offense variables, and criminal recidivism. These results should not be mistaken as entailing predictive power on individual level (71, 72). However, these group level associations can be of importance in identifying potential risk and protective factors among offenders with ID, in future attempts to develop prediction models of criminal recidivism in this specific population. In addition, while static risk assessments are important in prediction, they are of slightly less use to clinicians in their aim to reduce risk and customize therapeutic interventions. In forensic psychiatric clinical practices, risk assessments serve partially as material for targeting treatment interventions, and thus, clinical and dynamic variables are of greater interest. Our findings are useful in order to tailor treatment programs and adapt adequate preventive measures among ID offenders.

Using data from the FPA, ID diagnoses in the current study were ascertained based on a structured assessment of intellectual level and adaptive behavior and thus in concordance with current diagnostic criteria (73–75). This is a strength since several previous studies on ID offenders suffer from methodological weaknesses including an uncertain definition of ID (47). Using register data, we were able to include 259 ID offenders, which is a considerable amount of individuals in similar research contexts. We were able to study reconvictions during a period of up to 17 years following a sentence to forensic psychiatric care, which addresses the well-known problem in ID offender research, namely, low frequency base-rates of outcome. However, using data from the FPA inevitably entails a selected population of offenders, since only offenders of serious crimes (where incarceration is an applicable sanction) are included. Consequently, our results cannot be generalized to offenders of less severe crimes. In addition, our study population consists of offenders sentenced to forensic psychiatric care in Sweden. However, different countries have different regulations concerning offenders with mental disorders. The Swedish judicial system deviates from many others by not practicing insanity defense legislation. Offenders with severe mental disorders are ascribed legal responsibility and sentences are imposed. Individuals in forensic psychiatric care in Sweden might consequently not be identical to forensic psychiatric populations in other countries, possibly hampering generalizability of results. However, the Swedish system is in accordance with the basic principle of most developed countries, suggesting that an individual who has committed a crime under the influence of a severe mental disorder should not be sentenced prison. We therefore have reason to believe that the Swedish forensic psychiatric population consists of substantially the same patient categories as forensic populations in other countries.

It is important to note that our results present data on recidivism following a sentence to forensic psychiatric care, and not following discharge. An important limitation of the study is that it does not consider time at risk. The main reason is that the eligible registers had inadequate data on discharge dates for individuals in forensic psychiatric care, since the Swedish Patient Register started register specific data for inpatient involuntary psychiatric care in 2010 (representative for the Swedish Patient Register, personal communication, 19 October 2021). This could bias the results in two ways. Firstly, the risk of criminal recidivism is supposedly lower during incarceration. Secondly, there might be a difference in duration of inpatient treatment between offenders with and without ID. Studies comparing the duration of stay in inpatient facilities for ID and non-ID offenders present diverging results (76, 77). However, according to the Swedish National Forensic Psychiatric Register [initiated in 2008 and reaching 96% coverage by 2010 (78)], among individuals sentenced to forensic psychiatric care in Sweden between 2009 and 2021, the median length of stay among individuals with ID equals the average in the entire group including all diagnoses (59 vs. 58 months) (79). It should be noted, that these register data are based on main diagnosis and do not consider comorbidity. An individual with psychosis or affective disorder and concurrent ID might therefore not be registered in the ID group. Offenders with ID have been suggested as presenting with more severe symptoms and lower levels of educational and economic resources, personal strengths and social support than offenders with other mental disorders (77), which could induce a higher level of treatment measures and support, influencing recidivism risk. Unfortunately, our data cannot determine if a longer duration of inpatient care contributed to a lower risk of reconviction among offenders with ID.

A limitation of the study, shared with most other studies on ID offenders, is the sample size. The limited sample of ID offenders hampers certain estimates, such as sub-analyses of clinical categories or female ID offenders. The observation that ID was not associated with increased or decreased recidivism risk among female offenders is of interest, however, data had insufficient statistical power and further studies would be needed to verify this finding.

Individuals with ID are entitled to specific support in Sweden, according to the Act concerning Support and Service for Persons with Certain Functional Impairments (32). This legislation guarantees support for people with extensive functional impairment, such as ID, ensuring that they receive good living conditions and the service and help they need in daily life. Since the majority of individuals who were diagnosed with ID during the FPA were not identified as having ID prior to the assessment (12), it is plausible that the diagnosis could effectuate extra support, both during inpatient care (counseling and daily activities) but especially following discharge (financial support, adapted housing, group home, personal assistance, and contact with habilitation services) which might influence the risk of criminal recidivism. Our finding that the risk of criminal recidivism was lower among male offenders with ID can therefore be regarded as aligned with the legislator’s intention.

The most crucial finding from the current study was that offenders with ID who were sentenced to forensic psychiatric care presented with lower risk of criminal recidivism compared to offenders with other mental disorders. This finding is contrary to data from the meta-analysis by Bonta et al. (14). It highlights the importance of adequate diagnostic terms and definitions, since Bonta et al. employed the term intellectual impairment, which includes, but is not specific to, ID.

Another important finding was that young age and male sex; factors associated with increased risk of offending and re-offending in offender populations, were not associated with increased risk of re-offending among individuals with ID. This is clinically relevant, as many risk assessment instruments include age and sex. The increased risk associated with low age and male sex is so well-established that it will presumably impact the clinical risk assessment, even if actuarial instruments are not used.

Adequate knowledge of recidivism risk and factors associated with increased or decreased risk in clinical subgroups of offenders, such as ID offenders, is of utmost importance to the treating psychiatrist in the forensic psychiatric setting, both in creating treatment plans, in risk assessment and in communication with the court and other representatives from the criminal justice system.

## Data availability statement

The datasets presented in this article are not readily available because the present study is based upon data from Swedish National Registers. Swedish Data Protection Laws and the Swedish Ethical Review Authority exert joint protection of register data. Study data is consequently not publicly available. Other researchers may, however, contact Statistics Sweden and the Swedish National Board of Health and Welfare to get access to data from included registers. Requests to access the datasets should be directed to Department of Medical Epidemiology and Biostatistics in Karolinska Institutet, internservice@meb.ki.se.

## Ethics statement

The studies involving human participants were reviewed and approved by the Regional Ethical Review Board in Stockholm (2017/2531-31/5). Written informed consent from the participants’ legal guardian/next of kin was not required to participate in this study in accordance with the national legislation and the institutional requirements.

## Author contributions

HL collected the data. HE and QC organized the database. HE performed the statistical analyses. HE analyzed the results supervised by TH and QC, and wrote the first draft of the manuscript. TH, HL, PA, and QC proposed manuscript revisions. All authors contributed to the design of the study and conclusion and finally read and approved the submitted version of the manuscript.
