# Supplementary material for: Criminal recidivism in offenders with and without intellectual disability sentenced to forensic psychiatric care in Sweden—A 17-year follow-up study
Source: Front Psychiatry. 2022 Sep 21;13:1011984. doi: 10.3389/fpsyt.2022.1011984 (PMC9533124; doi:10.3389/fpsyt.2022.1011984)
Supplement: Supplementary file 1 [file Table_1.DOCX]

**Supplementary Table I.**
DSM-IV codes for diagnostic categories.

| **Diagnosis** | **DSM-IV-code(s)^a^** |
| --- | --- |
| ID | 317, 318, 319 |
| ADHD | 314 |
| ASD | 299 |
| Schizophrenia | 295 except 295.70 |
| Personality disorders | 301 |
| Alcohol use disorders | 291, 303, 305.00 |
| Drug use disorders | 292, 304, 305 except 305.00 |
| Sexual disorders | 302 except 302.6 and 302.85 |

ID: Intellectual Disability; ASD: Autism spectrum disorder

^a^ Three-digit codes include all subtypes and specifiers included in that code (four, five or six digits) unless other specified.
