# Supplementary material for: Criminal recidivism in offenders with and without intellectual disability sentenced to forensic psychiatric care in Sweden—A 17-year follow-up study
Source: Front Psychiatry. 2022 Sep 21;13:1011984. doi: 10.3389/fpsyt.2022.1011984 (PMC9533124; doi:10.3389/fpsyt.2022.1011984)
Supplement: Supplementary file 2 [file Table_2.DOCX]

**Supplementary Table II.**

Hazard ratios (HR) for criminal recidivism in offenders with ID compared to offenders without ID. Stratification for sex.

|  | Unadjusted HR | HR, model 1^a^ | HR, model 2^b^ |
| --- | --- | --- | --- |
| ID male (n=205) | 0.8 (0.7-1.1) | 0.8 (0.6-1.0)* | 0.8 (0.6-1.0) |
| Non-ID male (n=2637) | Reference | | |
| ID female (n=54) | 1.1 (0.6-1.9) | 1.0 (0.5-1.5) | 0.8 (0.4-1.5) |
| Non-ID female (n= 469) | Reference | | |

^*^ p <0.05 ^a^ Adjusted for age, previous criminal convictions and parental educational level
^b^ Adjusted for age, previous criminal convictions, parental educational level and concurrent clinical diagnoses (schizophrenia, personality disorders, ADHD, ASD, drug use disorder, alcohol use disorder and sexual disorder)
