# Supplementary material for: Criminal recidivism in offenders with and without intellectual disability sentenced to forensic psychiatric care in Sweden—A 17-year follow-up study
Source: Front Psychiatry. 2022 Sep 21;13:1011984. doi: 10.3389/fpsyt.2022.1011984 (PMC9533124; doi:10.3389/fpsyt.2022.1011984)
Supplement: Supplementary file 3 [file Table_3.DOCX]

**Supplementary Table III.**
Estimated hazard ratios for reconvictions comparing individuals with and without ID, with and without the psychiatric diagnoses ADHD, ASD, personality disorder, alcohol use disorder, drug use disorder, schizophrenia and sexual disorder. Hazard ratios are adjusted for potential confounders (age, sex, previous convictions and parental educational level).

| **Offenders sentenced to forensic psychiatric care (n=3365)** | **Any criminal reconviction** | | | | |
| --- | --- | --- | --- | --- | --- |
|  |  |  |  |  |  |
| **Exposure category** | **Events** | **Cumulative incidence (%)** | **Person years** | **Incidence rate^a^ (95% CI)** | **Hazard ratio (95% CI)** |
| **ADHD** | | | | | |
| ID + ADHD (n=21) | 8 | 38.1 | 108 | 73.9 (72.5-75.7) | 1.2 (0.6-2.4) |
| ID + non ADHD (n=238) | 66 | 27.7 | 1552 | 42.5 (42.2-42.8) | 0.8 (0.6-1.1) |
| Non ID + ADHD (n=123) | 58 | 47.2 | 574 | 101.0 (100.3-101.8) | 1.7 (1.3-2.2) |
| Non ID + non ADHD (n=2983) | 967 | 32.4 | 19527 | 49.5 (49.4-49.6) | Reference |
| **ASD** | | | | | |
| ID + ASD (n=66) | 16 | 24.2 | 342 | 46.7 (46.1-47.5) | 0.8 (0.5-1.3) |
| ID + non ASD (n=193) | 58 | 30.1 | 1317 | 44.0 (43.7-44.4) | 0.8 (0.6-1.0) |
| Non ID + ASD (n=313) | 63 | 20.1 | 1914 | 32.9 (32.7-33.2) | 0.6 (0.5-0.8) |
| Non ID + non ASD (n=2793) | 962 | 34.4 | 18187 | 52.9 (52.8-53.0) | Reference |
| **Personality disorder (pd)** | | | | | |
| ID + pd (n=55) | 16 | 29.1 | 442 | 36.2 (35.6-36.8) | 0.7 (0.4-1.2) |
| ID + non pd (n=204) | 58 | 28.4 | 1218 | 47.6 (47.2-48.0) | 0.9 (0.7-1.2) |
| Non ID + pd (n=817) | 369 | 45.2 | 5599 | 65.9 (65.7-66.1) | 1.3 (1.2-1.5) |
| Non ID + non pd  (n= 2289) | 656 | 28.6 | 14502 | 45.2 (45.1-45.3) | Reference |

**Supplementary Table III (cont.).**

| **Offenders sentenced to forensic psychiatric care (n=3365)** | **Any criminal reconviction** | | | | |
| --- | --- | --- | --- | --- | --- |
|  |  |  |  |  |  |
| **Exposure category** | **Events** | **Cumulative incidence (%)** | **Person years** | **Incidence rate^a^ (95% CI)** | **Hazard ratio (95% CI)** |
| **Alcohol use disorder (alc)** | | | | | |
| ID + alc (n=50) | 17 | 34.0 | 377 | 45.1  (44.4-45.8) | 0.9 (0.5-1.4) |
| ID + non alc (n=209) | 57 | 27.3 | 1283 | 44.4  (44.1-44.8) | 0.8 (0.6-1.1) |
| Non ID + alc (n=627) | 239 | 38.1 | 4333 | 55.2  (54.9-55.4) | 1.1 (1.0-1.3) |
| Non ID + non alc (n=2479) | 786 | 31.7 | 15768 | 49.8  (49.7-50.0) | Reference |
| **Drug use disorder (drug)** | | | | | |
| ID + drug (n=27) | 12 | 44.4 | 148 | 80.9  (79.7-82.5) | 1.5 (0.9-2.8) |
| ID + non drug (n=232) | 62 | 26.7 | 1511 | 41.0  (40.7-41.4) | 0.9 (0.7-1.2) |
| Non ID + drug (n=949) | 455 | 47.9 | 5101 | 89.2  (88.9-89.4) | 1.9 (1.6-2.1) |
| Non ID + non drug (n=2157) | 570 | 26.4 | 15000 | 38.0  (37.9-38.1) | Reference |
| **Schizophrenia (scz)** | | | | | |
| ID + scz (n=17) | 1 | 5.9 | 86 | 11.6  (10.9-12.4) | 0.2 (0.0-1.4) |
| ID + non scz (n=242) | 73 | 30.2 | 1574 | 46.4  (46.0-46.7) | 0.8 (0.6-1.0) |
| Non ID + scz (n=929) | 260 | 28.0 | 6476 | 40.1  (40.0-40.3) | 0.7 (0.6-0.8) |
| Non ID + non scz  (n= 2177) | 765 | 35.1 | 13625 | 56.1  (56.0-56.3) | Reference |
| **Sexual disorder (sex dis)** | | | | | |
| ID + sex dis (n=15) | 1 | 6.7 | 161 | 6.2 (5.8-6.6) | 0.1 (0.0-0.8) |
| ID + non sex dis  (n=244) | 73 | 30.0 | 1499 | 48.7  (48.4-49.0) | 0.9 (0.7-1.1) |
| Non ID + sex dis  (n=76) | 22 | 28.9 | 674 | 32.6  (32.2-33.1) | 0.7 (0.4-1.0) |
| Non ID + non sex dis (n=3030) | 1003 | 33.1 | 19427 | 51.6  (51.5-51.7) | Reference |
